# The Reactome Knowledgebase 2026

**DOI:** 10.1093/nar/gkaf1223

**Published:** 2025-11-18

**Authors:** Eliot Ragueneau, Chuqiao Gong, Pierre Sinquin, Cristoffer Sevilla, Deidre Beavers, Alexander Grentner, Johannes Griss, Gregory F J Hogue, Nancy T Li, Lisa Matthews, Bruce May, Marija Milacic, Helia Mohammadi, Robert Petryszak, Karen Rothfels, Veronica Shamovsky, Ralf Stephan, Krishna Tiwari, Joel Weiser, Adam Wright, Marc Gillespie, Guanming Wu, Lincoln Stein, Henning Hermjakob, Peter D’Eustachio

**Affiliations:** European Molecular Biology Laboratory, European Bioinformatics Institute (EMBL–EBI), Wellcome Genome Campus, Hinxton, Cambridgeshire CB10 1SD, United Kingdom; European Molecular Biology Laboratory, European Bioinformatics Institute (EMBL–EBI), Wellcome Genome Campus, Hinxton, Cambridgeshire CB10 1SD, United Kingdom; European Molecular Biology Laboratory, European Bioinformatics Institute (EMBL–EBI), Wellcome Genome Campus, Hinxton, Cambridgeshire CB10 1SD, United Kingdom; European Molecular Biology Laboratory, European Bioinformatics Institute (EMBL–EBI), Wellcome Genome Campus, Hinxton, Cambridgeshire CB10 1SD, United Kingdom; Division of Oncological Sciences, Knight Cancer Institute, Oregon Health and Science University, Portland, OR 97239, United States; European Molecular Biology Laboratory, European Bioinformatics Institute (EMBL–EBI), Wellcome Genome Campus, Hinxton, Cambridgeshire CB10 1SD, United Kingdom; Department of Dermatology, Medical University of Vienna, 1090 Vienna, Austria; European Molecular Biology Laboratory, European Bioinformatics Institute (EMBL–EBI), Wellcome Genome Campus, Hinxton, Cambridgeshire CB10 1SD, United Kingdom; Department of Dermatology, Medical University of Vienna, 1090 Vienna, Austria; Ontario Institute for Cancer Research, Toronto, ON M5G0A3, Canada; Ontario Institute for Cancer Research, Toronto, ON M5G0A3, Canada; NYU Grossman School of Medicine, New York University, New York, NY 10016, United States; Ontario Institute for Cancer Research, Toronto, ON M5G0A3, Canada; Ontario Institute for Cancer Research, Toronto, ON M5G0A3, Canada; Ontario Institute for Cancer Research, Toronto, ON M5G0A3, Canada; Department of Molecular Genetics, University of Toronto, Toronto, ON M5S 1A1, Canada; Division of Oncological Sciences, Knight Cancer Institute, Oregon Health and Science University, Portland, OR 97239, United States; Ontario Institute for Cancer Research, Toronto, ON M5G0A3, Canada; NYU Grossman School of Medicine, New York University, New York, NY 10016, United States; Ontario Institute for Cancer Research, Toronto, ON M5G0A3, Canada; Institute for Globally Distributed Open Research and Education (IGDORE); European Molecular Biology Laboratory, European Bioinformatics Institute (EMBL–EBI), Wellcome Genome Campus, Hinxton, Cambridgeshire CB10 1SD, United Kingdom; Open Targets, Wellcome Genome Campus, Hinxton, Cambridgeshire CB10 1SD, United Kingdom; Ontario Institute for Cancer Research, Toronto, ON M5G0A3, Canada; Ontario Institute for Cancer Research, Toronto, ON M5G0A3, Canada; Ontario Institute for Cancer Research, Toronto, ON M5G0A3, Canada; College of Pharmacy and Health Sciences, St. John’s University, Queens, NY 11439, United States; Division of Oncological Sciences, Knight Cancer Institute, Oregon Health and Science University, Portland, OR 97239, United States; Ontario Institute for Cancer Research, Toronto, ON M5G0A3, Canada; Department of Molecular Genetics, University of Toronto, Toronto, ON M5S 1A1, Canada; European Molecular Biology Laboratory, European Bioinformatics Institute (EMBL–EBI), Wellcome Genome Campus, Hinxton, Cambridgeshire CB10 1SD, United Kingdom; NYU Grossman School of Medicine, New York University, New York, NY 10016, United States

## Abstract

The Reactome Knowledgebase (https://reactome.org) is a freely accessible, expert-curated, open-source, and open-data resource that describes human biology in molecular detail. It spans normal physiology as well as disease mechanisms, including the impact of genetic variation and drug action. Reactome content is continuously expanded and revised, with automated workflows now monitoring retracted publications to maintain data integrity. To meet the needs of a growing user base, Reactome has launched a redesigned Angular-based interface with enhanced accessibility, modular architecture, and a hierarchy of visualization tools: ReacFoam for global pathway overviews, enhanced high-level diagrams for intuitive navigation, and redesigned entity level views (ELVs) enriched with chemical structures, animated protein models, and a new “compare mode” to contrast normal and disease states. New analysis tools support multi-omics integration and customizable visualizations. Recent innovations include the React-to-me chatbot for natural language interaction, community-driven tutorials, and an open Figma icon library. Reactome’s sustainability and compliance with FAIR data principles were recently recognized with CoreTrustSeal certification and its designation as a Global Core Biodata and ELIXIR resource, reinforcing its role as a trusted global knowledgebase.

## Graphical Abstract

**Graphical Abstract:**
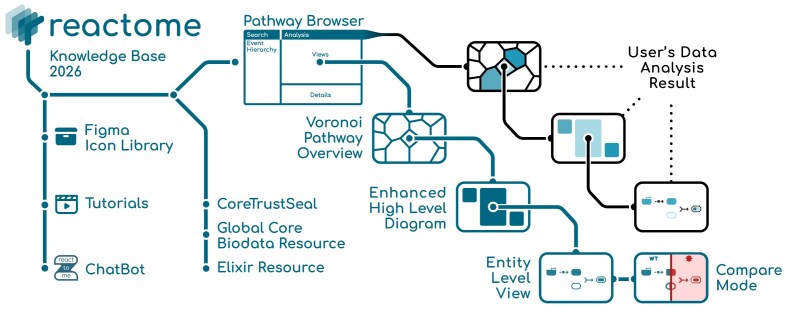

## Introduction

At the cellular level, biological processes can be represented by networks of molecular reactions that drive signal transduction, transport, DNA replication, protein synthesis, and intermediary metabolism. Various online resources capture this information at the level of individual reactions, such as Rhea [1], or at the level of reaction sequences covering many domains of biology, such as KEGG [2] or MetaCyc [3]. The Reactome Knowledgebase is distinctive in focusing its manual annotation effort on a single species, *Homo sapiens*, and applying a single consistent data model across all domains of biology. Processes are systematically described in molecular detail to generate an ordered network of molecular transformations, resulting in an extended version of a classic metabolic map generally compliant with the SBGN process description standard [4]. The Reactome Knowledgebase systematically links human proteins to their molecular functions, providing a resource that is both a textbook of biological processes and a tool for discovering novel functional relationships in data such as tissue-, cell-, or physiological state-specific gene expression, catalogs of somatic mutations in tumor cells, or likely effects of drugs based on their known interactions with proteins across annotated pathways.

Reactome (version 94; September 2025) has entries for 11 630 gene products involved in 16 002 reactions annotated from 41 373 literature references (Table 1). These reactions are grouped into 2825 pathways (e.g. Interleukin-15 signaling) collected under 29 superpathways (e.g. immune system) that describe normal cellular functions. A “Disease” superpathway includes abnormal processes driven by germline and somatic mutations, and ones due to the actions of genes of infectious bacteria, viruses, and parasites. Genetic disease annotations cover 5507 variant proteins and posttranslationally modified forms of them, derived from 392 human gene products. Infectious disease pathways include the effects of bacterial toxins, aspects of infection by Leishmania, Listeria and Mycobacteria, and viral infections [5] mediated by influenza, HIV, human cytomegalovirus, respiratory syncytial virus, and SARS-CoV-1 and -2. In addition, Reactome describes the modulating effects of 1070 drugs on both normal and disease processes.

**Table 1.** Reactome content; version 86 (September 2023) versus 94 (September 2025)

Data type	Release 86	Release 94	Change
Human proteins	11 148	11 630	482
Proteoforms	30 338	31 991	1653
Chemicals	2025	2176	151
Reactions	14 803	16 002	1199
Human disease proteins	354	392	38
Disease variants	4919	5507	588
Chemical drugs	952	975	23
Protein drugs	86	95	9
Literature references	37 156	41 373	4217

“Human proteins” is the number of human UniProt entries (not counting isoforms) annotated in Reactome. Each may be represented as multiple proteoforms to account for covalent modifications and subcellular locations. “Disease proteins” are ones whose germline or somatic variation gives rise to proteins with altered, pathogenic functions. “Disease variants” is the total number of such variant alleles annotated in Reactome.

## Managing retracted publications

A growing concern for Reactome and other data repositories based on published literature is the high and rapidly increasing frequency of retracted papers, e.g. [6]. We have developed scripts [7] to review all publications we have cited to identify ones whose PubMed “publication type” is “retracted publication” or which are listed in Retraction Watch Data [6]. A total of 62 papers from the Retraction Watch database were identified in Reactome; of these, 51 were deemed unusable. In reviewing 90 affected reactions, 6 were unaffected, 66 required revision, and 18 were removed. Additionally, 129 pathway and reaction descriptions were examined, resulting in 108 revisions. This impact (62 of 39 806 literature references; 18 of 15 591 reactions) is remarkably low. Two longstanding editorial policies may explain it. First, we do not provide comprehensive annotations of all relevant literature but rather identify a minimum set of authoritative publications to describe the current expert consensus view of a biological process, verified by external reviewers. Second, whenever possible, we rely on two or more independent expert views to annotate a reaction.

## Annotating human biology; a new user interface

Even with a steadily growing body of annotations (Table 1), Reactome captures only a fraction of human molecular physiology. This coverage already supports useful analyses, e.g. to identify gene products whose expression is altered in response to an environmental stress and link them to specific processes perturbed as a result (e.g. [8]) or to develop biologically informed neural networks to enhance proteomic biomarker discovery in disease [9, 10]. Over the past 20 years, we have built a variety of tools to enable data visualization and analysis at multiple levels of granularity. To continue to function effectively, we need a data scheme and analysis tools that scale well as our coverage expands and that adapt to address novel computational needs arising from technological advances in experimental research.

To meet these challenges, we have redeveloped our user interface (UI) and user experience using Angular [11] to ensure maintainability and facilitate development. The new UI is themed by a few CSS variables that establish a consistent visual identity for Reactome while facilitating future revisions, extensions, and UI customization. For instance, the UI enhances accessibility of Reactome by allowing users to adjust its behavior to meet their specific needs, e.g. color palettes suitable for color-blindness and introduction of light and dark modes to reduce eye fatigue (Supplementary Fig. S1). It also supports the development of widgets that enable partner organizations (https://reactome.org/community/partners) to embed Reactome views within their websites without compromising their own designs.

This new architecture is available now in a beta version at https://reactome.org/beta/PathwayBrowser and will become the production version in the first half of 2026. Feedback is welcome through the Google form https://forms.gle/gEpbmg9q7j1XHXDVA, or as a GitHub Issue https://github.com/reactome/PathwayBrowser/issues.

## A hierarchy of pathway visualization tools

We describe this new web environment, descending from the entirety of human reaction space to the molecular details of single reactions.

### ReacFoam overview of reaction space

**Figure 1.**
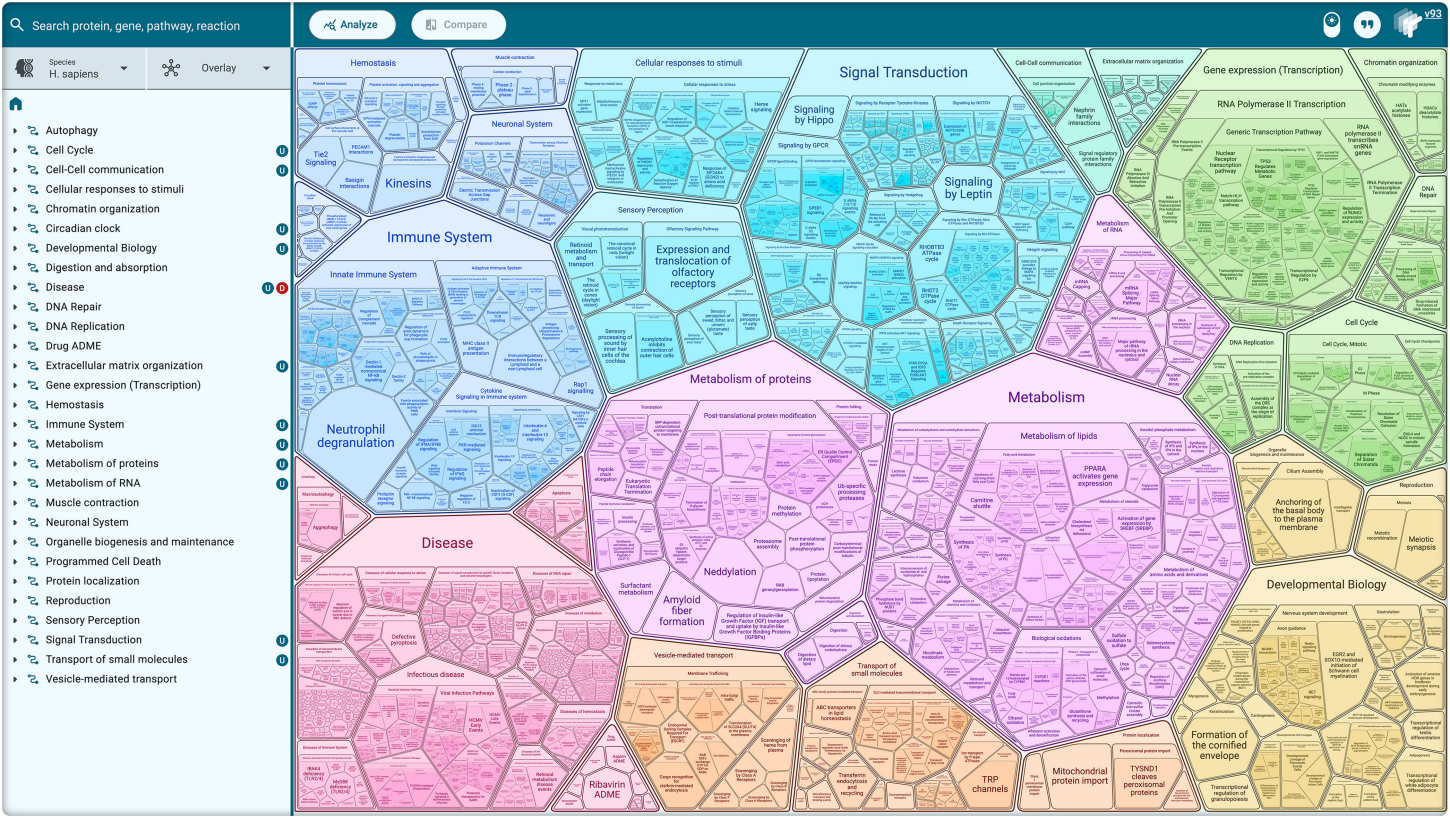
“ReacFoam” overview of all human pathways (ReacFoam view). The left panel of the web page displays the entire event hierarchy. The right panel represents it as a Voronoi diagram, organized to group related pathways as indicated by the colors, and sized to show the relative content of each pathway polygon.

To effectively support top–down exploration of Reactome, we developed the “ReacFoam” view [12], based on Voronoi diagrams built with the FoamTree library [13] (Fig. 1). This display presents a human-friendly genome-wide visualization of pathway space, organized hierarchically. Each polygon represents a pathway and can contain subpathways. Their sizes are determined by the number of molecules in the pathway and their color by the category of their top-level pathway. The layout flows to adjust to viewport size but preserves relative positions of polygons to ensure a stable ReacFoam appearance across platforms. The ReacFoam display can be navigated by zooming and clicking on items of interest.

To the left of the ReacFoam visualization, the Event Hierarchy displays the same hierarchical structure but extends it further by unfurling to show reactions. Drop-down tabs above the hierarchy allow the user to control overlay features in the display and to display computationally inferred pathways for model organisms. Tags on the right side of the hierarchy indicate the status of each pathway (new/revised; disease/normal).

### Pathway illustrations (EHLDs)

**Figure 2.**
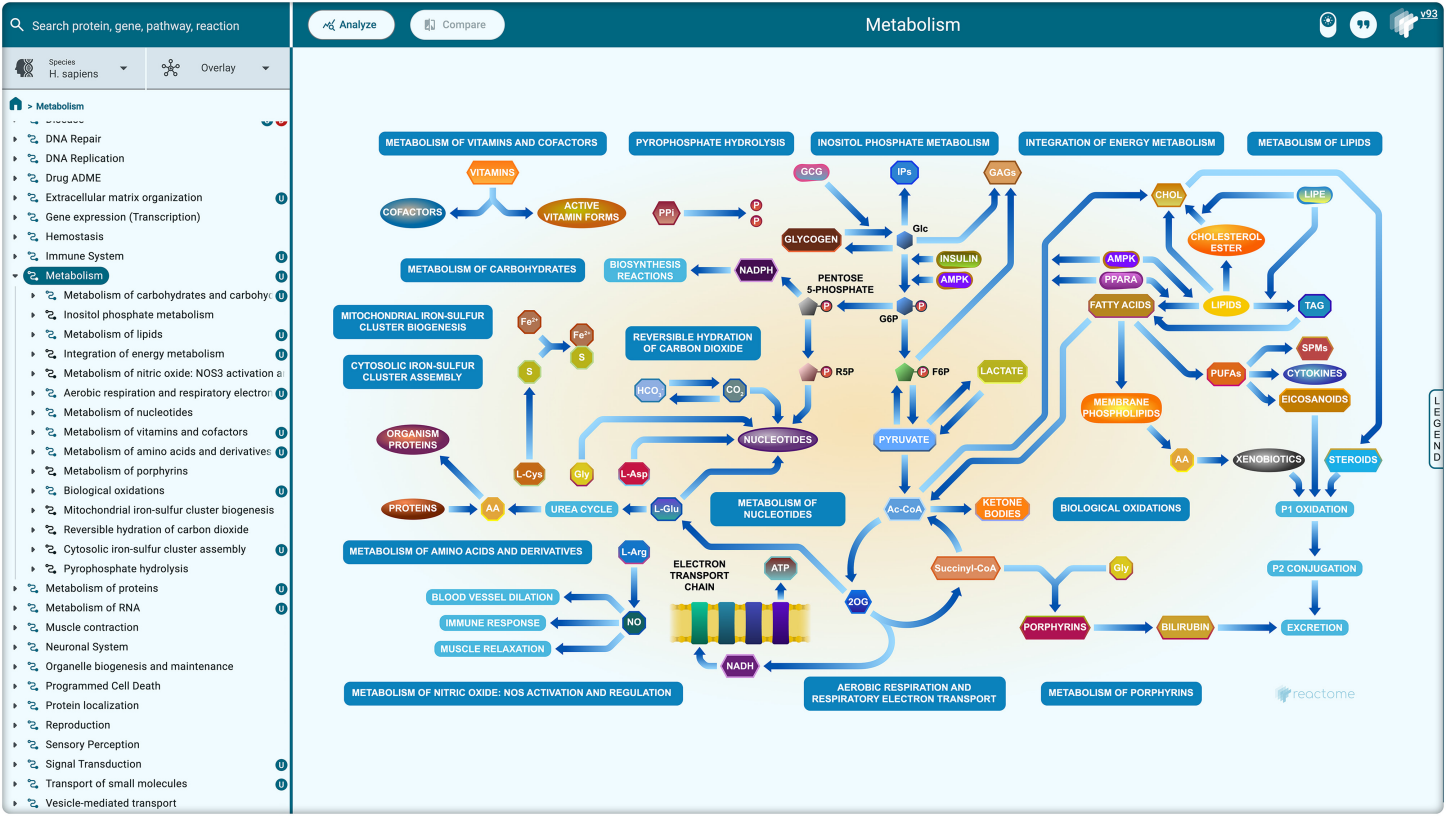
EHLD view of the metabolism superpathway. The event hierarchy (left) is unfurled to reveal subpathways of metabolism. The EHLD represents them as sections of a textbook-style overview.

Navigating to a high-level pathway reveals an enhanced high level diagram (EHLD), a vector graphic that provides a textbook-style overview of the whole pathway (Fig. 2) [14], All 29 top-level pathways (e.g. metabolism, signal transduction) and 183 of the other 205 high-level pathways are now represented as EHLDs (release 94, September 2025).

EHLDs are composed of icons that represent molecular entities, cells, and tissues, custom-made by Reactome’s scientific illustrator. They are released to the public under the CC BY 4.0 license to encourage reuse of the icons in other contexts (https://reactome.org/icon-lib). We have also started to release complex EHLD illustration backgrounds through the Icon Library, with 18 now available. These are useful as frameworks that others can use to lay out their own diagrams,

The 2356 distinct icons from the library are also now freely available in Figma, which facilitates the design of scientific illustrations by making icons easily accessible and searchable directly within a free design ecosystem. The Figma library is available at https://www.figma.com/community/file/1504140227575930077. Also, thanks to Figma’s component system, designers will be notified if any of the icons they are using have been updated in the Reactome Library, and can update their illustrations automatically.

### Individual pathway diagrams (entity level views)

**Figure 3.**
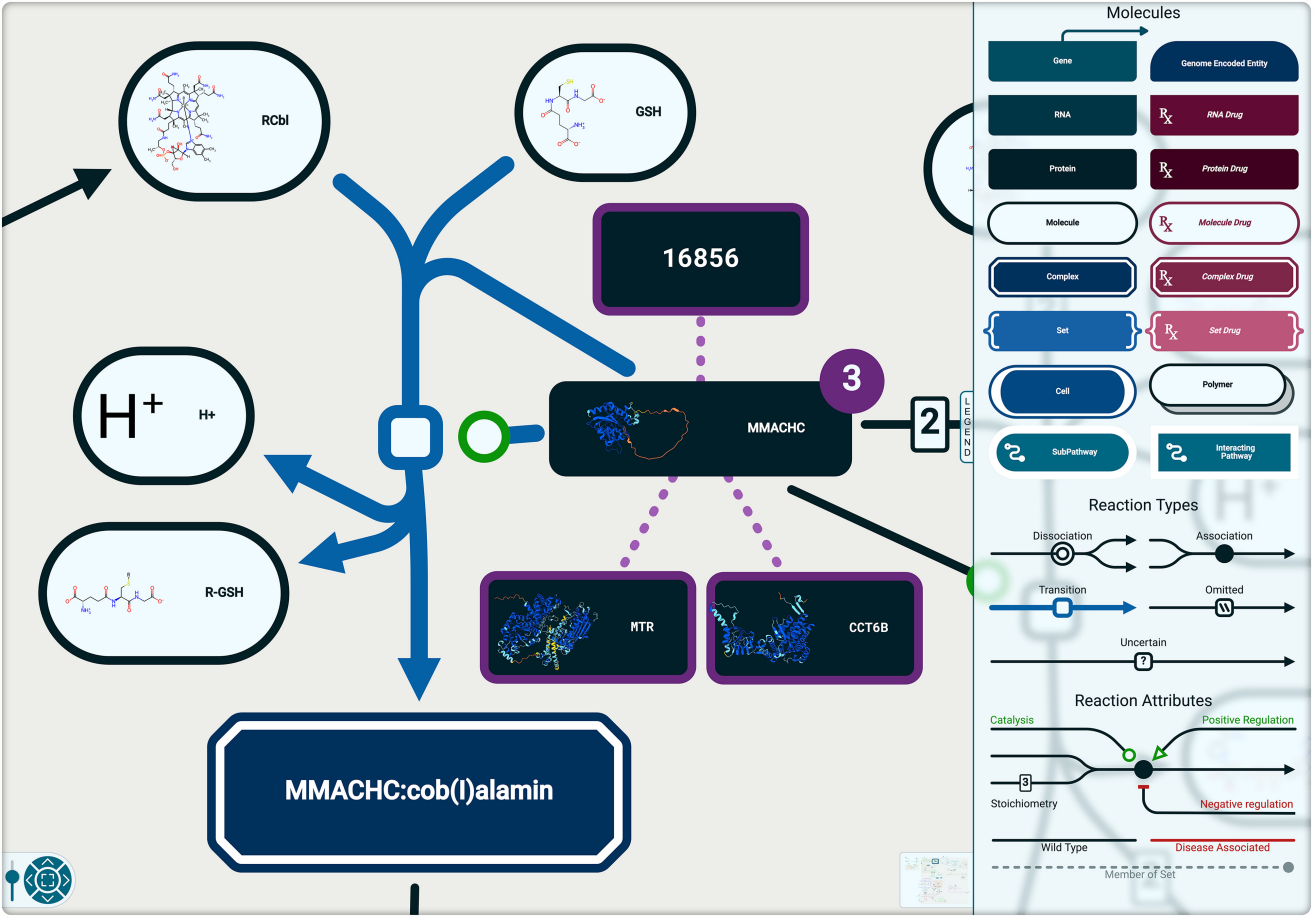
View of a single reaction in an ELV (R-HSA-3095889-MMACHC dealkylates RCbl). Zoomed in, the icons for protein and chemical participants in the reaction show structure details; the legend on the right provides a key to entity and event types (legend details in Supplementary data).

ELVs (Fig. 3) show the molecular details of individual reactions and connections among them. The angles in physical entities and edges have been rounded to indicate reaction direction, and a more homogenous color palette and more coherent internal logic have been implemented while staying true to the systems biology graphical notation (SBGN) standard [4]. To implement this new design, we used the open-source application ecosystem cytoscape.js [15] to accelerate development. We have contributed a new plugin called reactome-cytoscape-style [16] and have developed and contributed features we needed, such as the round edge types.

The legend for the ELVs is now a draggable panel on the right. Inside, every diagram element type is represented by an icon. Shape and color have been used to visually group similar entities and edges (Supplementary Fig. S2). These icons are interactive: selecting the protein icon, for instance, will highlight all proteins in the diagram. Similarly, hovering over a diagram element will highlight its type in the legend.

As users zoom into the view, subpathways’ color distinctions disappear while chemical and protein structures appear. Chemicals’ structures are represented by images imported from ChEBI [17], while proteins’ structures are represented by prerendered rotating videos. Most of the protein structures are imported from AlphaFold Protein Structure Database [18, 19] due to its full-length coverage of each protein sequence. When not available, the most representative structures are chosen based on 3D-Beacons [20] and PDBe APIs [21].

Structures are rendered within Mol* [22] into videos with transparent background, legible in both light and dark modes (.webm with libvpx-vp9 encoder for Chrome and Firefox, .mov with hevc_videotoolbox encoder for Safari) and stored in AWS S3 to be accessible not only to the new diagrams but also to anyone wanting to integrate many animated 3D structures at once on a webpage but also to anyone wanting to integrate many animated 3D structures at once on a webpage. Videos are available at https://download.reactome.org/structures/, followed by a UniProt ID and the extension .mov or .webm (e.g. https://download.reactome.org/structures/Q9Y4U1.webm). The corresponding .json file provides information on the source structure used in each video.

Users can overlay protein–protein and other pairwise interaction data on ELV diagrams by selecting an overlay resource in the left panel. If a molecule has interactors, or other types of associated data, a bubble appears in the top right corner of its icon that displays the number of associated elements. Clicking expands the bubble into a list of the top 20 associated elements.

### Accessing details of events and physical entities

When users select an event or a physical entity, all underlying data appear in the “Details” tab of the bottom panel. Rapid growth in the kinds and amounts of relevant underlying data has driven the development of a new “Details” panel that displays information efficiently, with minimum user navigation. This display was implemented by flattening the core sections into a long scrollable document, which can easily be browsed by using the Table of Contents on the right-hand side to directly access the sections of interest. Deeply nested elements can be unfurled stepwise or entirely with a single click.

New sections have also been introduced, including structures of chemicals and proteins, as interactive widgets for 3D structures. When a Reactome reaction has a link to its RHEA [1] counterpart, structural details of the reaction are shown using its widget.

Every protein, DNA or RNA molecule, and small chemical in Reactome is mapped to a reference entity from UniProt, Ensembl, or ChEBI, respectively. Specific forms and localization of these molecules (e.g. a chemically modified protein localized to the cytosol) are the actors of reactions and are mapped to these reference entities. Reactome now offers a summary feature that provides users an overview of all actions of the reference entity. By default, Reactome avoids the inclusion of potentially complex disease-related data into this summary, but users can adjust this behavior. Users can access these summaries by using the select button next to the external reference of proteins and other molecules.

### Search

The search bar, now in the upper left corner, also benefits from this new summary definition. As noted, search results are now merged by reference entity to summarise all their actions in a single line, which users can then inspect further to see all pathways where those molecules act. Additionally, the search functionality has improved filters and clarifies the distinction between local and global search results. Every search result, regardless of its nature, can be flagged to increase its visibility across all views by highlighting the element itself and the containers of those elements with a distinctive bright pink color (Supplementary Fig. S3).

### Analysis

Reactome offers diverse analysis methods for users to project experimental omics data across all pathway space, enabling the identification of pathways of interest. The methods accept diverse input datasets, ranging from a simple list of genes of interest, e.g. mutated genes identified in a tumor specimen for qualitative enrichment analysis, to a multisample multi-omics dataset for quantitative enrichment analysis (ReactomeGSA).

**Figure 4.**
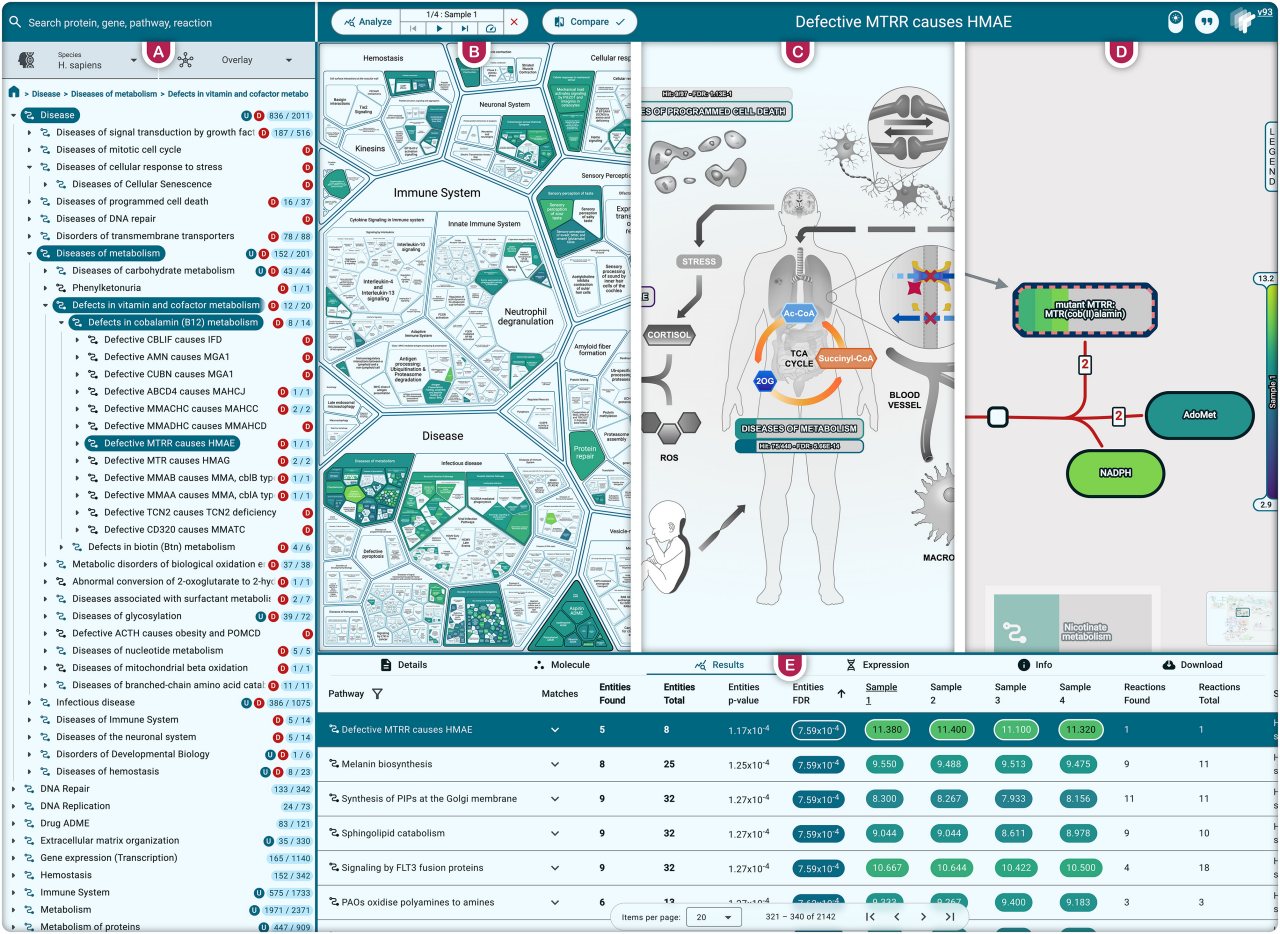
Composite screenshot of analysis results. These are visualized alternatively in (**A**) the Event Hierarchy, (**B**) ReacFoam, (**C**) an EHLD, (**D**) an ELVs, and (**E**) Results table. The results shown are from a qualitative enrichment analysis of the “Metabolomics” example. Panel (B) is taken from the overview, panel (C) from R-HSA-1643685, and panels (A) ,(D), and (E) are taken from R-HSA-3359467.

Users can launch analysis and control the visualized sample using the analysis button placed on the top bar (Fig. 4B). Data submission has been simplified, notably by embedding a table editor [23] and by providing animations to show the effects of analysis options.

Analysis results can be displayed in each UI view (Fig. 4). The event hierarchy (Fig. 4A) displays the number of reactions found in each pathway. The key analysis metrics [i.e. false discovery rate (FDR), expression, etc.] are mapped to a customisable color scale in the detailed reaction diagram (right-hand side of Fig. 4D). This color scale is then applied to statistically significant pathways in the ReacFoam (Fig. 4B) and EHLD (Fig. 4C), while nonsignificant ones are grayed out. In the ELV (Fig. 4D), the flattened components of complexes, sets, and cell markers are individually colored. Their dedicated sections are arranged according to the key metric, producing a continuous color gradient that reflects the overall status of the multicomponent entity.

The results table (Fig. 4E) lists all matching pathways, ranked from most to least significant. Results for each analyzed sample are displayed in a separate column representing the average value of the sample metric among pathway participants. Pathways are sorted by data in any column by clicking on its header. The results can be filtered in the table according to different characteristics, like the FDR or the current sample value. A new “pathway grouping” filter strips all higher-level pathways from the results display, leaving only the ELV-level ones. Combined with the new “Focus mode,” which enables pathway filtering to influence the ReacFoam structure (Supplementary Fig. S4A versus B), this “Pathway grouping” filter also collapses higher-level pathways into a single one containing all ELVs (Supplementary Fig. S4C).

### Compare mode

**Figure 5.**
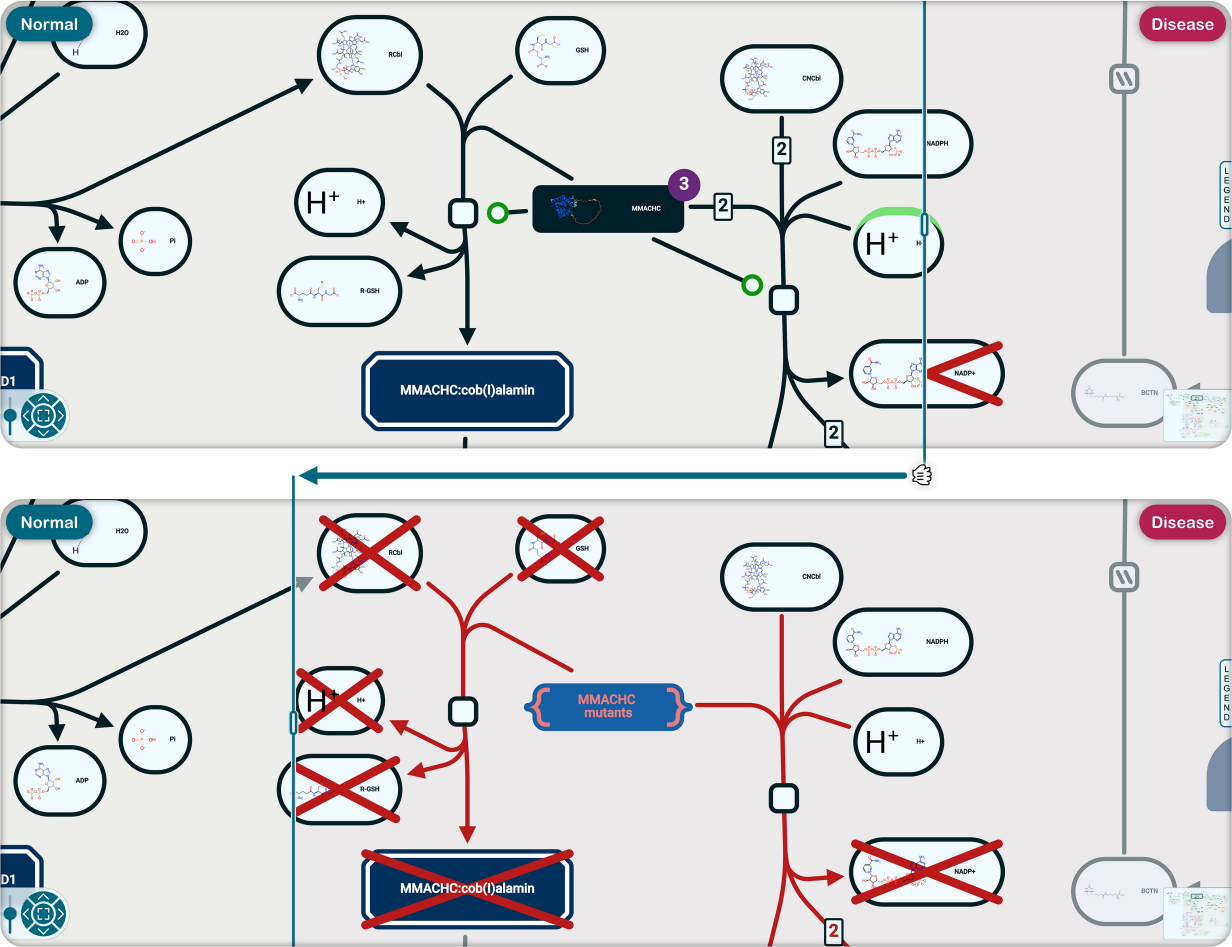
Mutations in MMACHC protein (R-HSA-3359474) disrupt two reactions of normal cobalamin metabolism (R-HSA-3095889 and R-HSA-3149519). The top panel shows the normal reactions; dragging the vertical border bar leftward reveals the details of the reactions disrupted by the mutations.

Some disease pathways represent losses and gains of function of proteins as variants of the corresponding normal pathway. A new visualization feature superimposes the two versions of the pathway in distinct layers, and a slide bar can be dragged to expose either one, enabling direct comparison of the two conditions (Fig. 5).

This comparison feature is currently implemented for normal/disease pathway pairs but could be extended to other comparisons, such as orthology projection.

## AI integration: React-to-me chatbot

To improve user accessibility, we released the React-to-Me AI chatbot in February 2025 (https://reactome.org/chat/), integrating Reactome’s pathway knowledge with AI, using retrieval-augmented generation (RAG) workflows to minimize AI hallucination. The chatbot enables users to query Reactome in natural language and receive detailed, citation-linked answers (together with warning that hallucinations remain possible). Evaluation through a structured survey of academic, clinical, and industry users indicated high satisfaction with scientific accuracy, source transparency, and ease of use. Future iterations will incorporate user feedback to further refine functionality.

We are using the same RAG workflows for internal curation purposes. Building on the results of a study done in 2022 evaluating the use of generative AI in the curation process (https://www.biorxiv.org/content/10.1101/2023.11.08.566195v1), we are now developing tools to assist in literature triage and relationship extraction, supporting the identification of unannotated genes, proposing pathway associations, and suggesting curation targets. All of these tools are aimed at filtering large bodies of potentially relevant material to identify the subset most useful for expert manual review and possible curation.

## Community: online tutorials

We have created a playlist, “Getting Started with Reactome,”of 1–3 minute videos, with English subtitles that can be translated into other languages, to introduce new users to Reactome’s main functionalities. The playlist provides an introduction to Reactome and walkthroughs of the pathway browser, main search, analysis tools, downloadable materials, and how to contribute to Reactome. The playlist is available on the Reactome Knowledgebase YouTube channel (https://youtube.com/playlist?list=PLLc8-qElSrCxhMsCVjYIO8OmO-Im_lQ6D&si=nDq9FPlZPMq7jGUR). Work is underway to revise these videos to align them with the features of the new website described above, coordinated with the planned full release of the website in the first half of 2026.

## Data availability

The CoreTrustSeal organization promotes sustainable and trustworthy data infrastructures by establishing requirements for trustworthy repositories, reviewing repositories, and certifying ones that meet the requirements [24]. In 2025, Reactome was awarded CoreTrustSeal certification, independently validating our sustainability, transparency, governance, and commitment to longterm preservation. As part of the certification process, Reactome has fully documented its digital preservation and sustainability plan (https://reactome.org/about/digital-preservation), and its curation and quality assurance standards (https://reactome.org/documentation/curator-guide). All Reactome data are available in various formats from our downloads page (https://reactome.org/download-data) and from Zenodo (https://zenodo.org/records/15731221), as is our users’ guide and related documentation (https://zenodo.org/records/15808136), and software for our pathway browser (https://doi.org/10.5281/zenodo.17379609) and React-to-me chatbot (https://doi.org/10.5281/zenodo.17379758), under terms that allow free reuse and redistribution. To enable users to track changes in our annotations over time, we have released a history tracking tool.

These materials enhance transparency for our community, aligning with FAIR principles and best practices in open science. Together with Reactome’s recognition as a Global Core Biodata Resource (https://zenodo.org/records/7468719) and an ELIXIR Core Data Resource [25], this certification positions Reactome among the world’s leading repositories of trusted biological knowledge, reinforcing confidence in its longterm reliability for users.

## Conclusions

The Reactome Knowledgebase detailing the molecular details of human biological processes continues to grow in size, scope, and integration with other resources. Here, we have described the implementation of a new website that accommodates this growth, enhances data visualization and navigation for human users, and provides easier access to data analysis tools. Additional new features include the Reacto-to-me chatbot trained on Reactome content and a dynamic set of tools to detect retractions of publications used as evidence in support of Reactome events.

## Supplementary Material

gkaf1223_Supplemental_File
