# Supplementary material for: The Reactome Knowledgebase 2026
Source: Nucleic Acids Res. 2025 Nov 18;54(D1):D673–81. doi: 10.1093/nar/gkaf1223 (PMC12807730; doi:10.1093/nar/gkaf1223)
Supplement: gkaf1223_Supplemental_File [file gkaf1223_supplemental_file.pdf]

SUPPLEMENTARY FIGURES AND LEGENDS

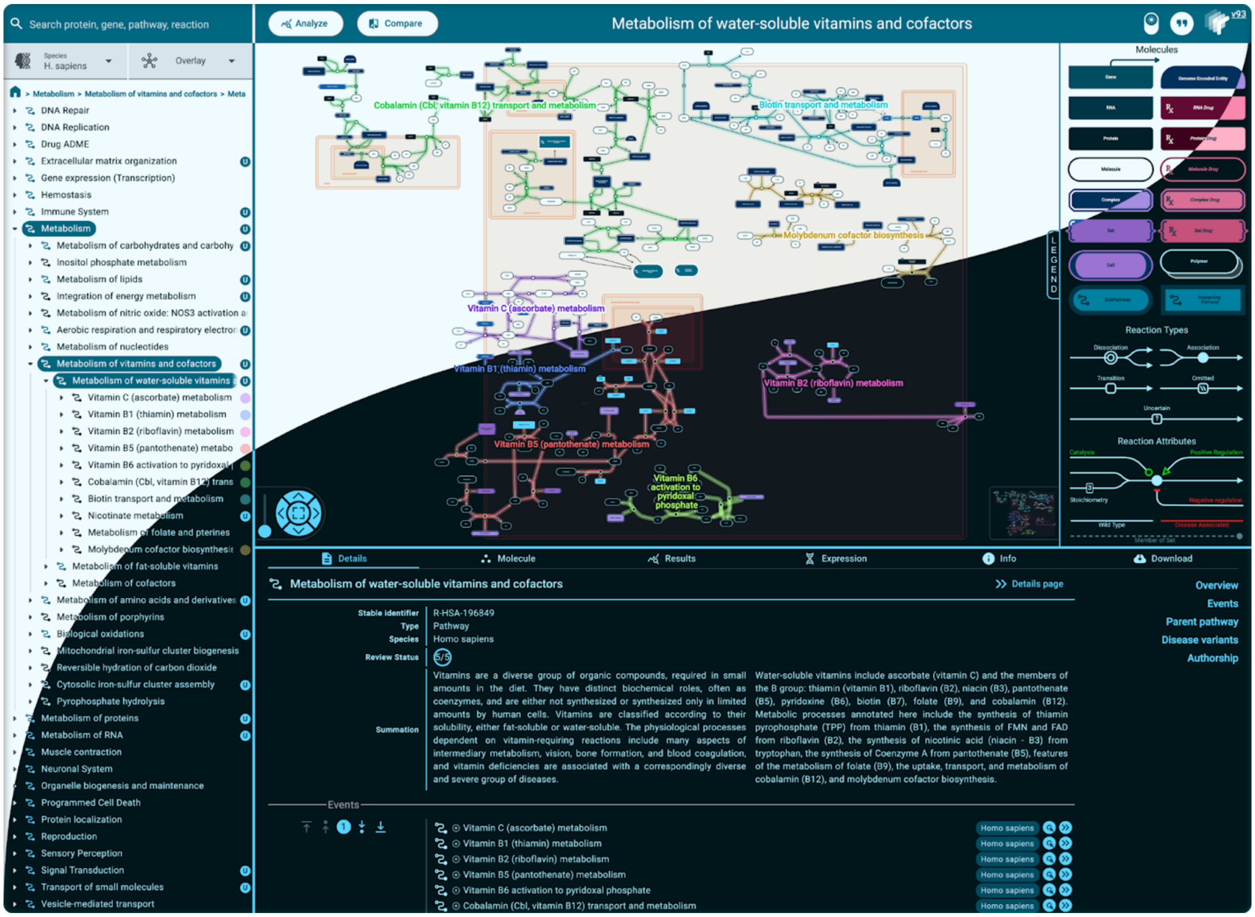

Supplementary Figure 1 – Light and dark display modes. A composite screenshot of the Entity-Level View (ELV) of the "Metabolism of water-soluble vitamins and cofactors" pathway ([R-HSA-196849](R-HSA-196849)) in the new browser interface, shows the effect of light and dark display modes. The "legend" tab at the right of the screen is opened (see also Supplementary Figure 2).

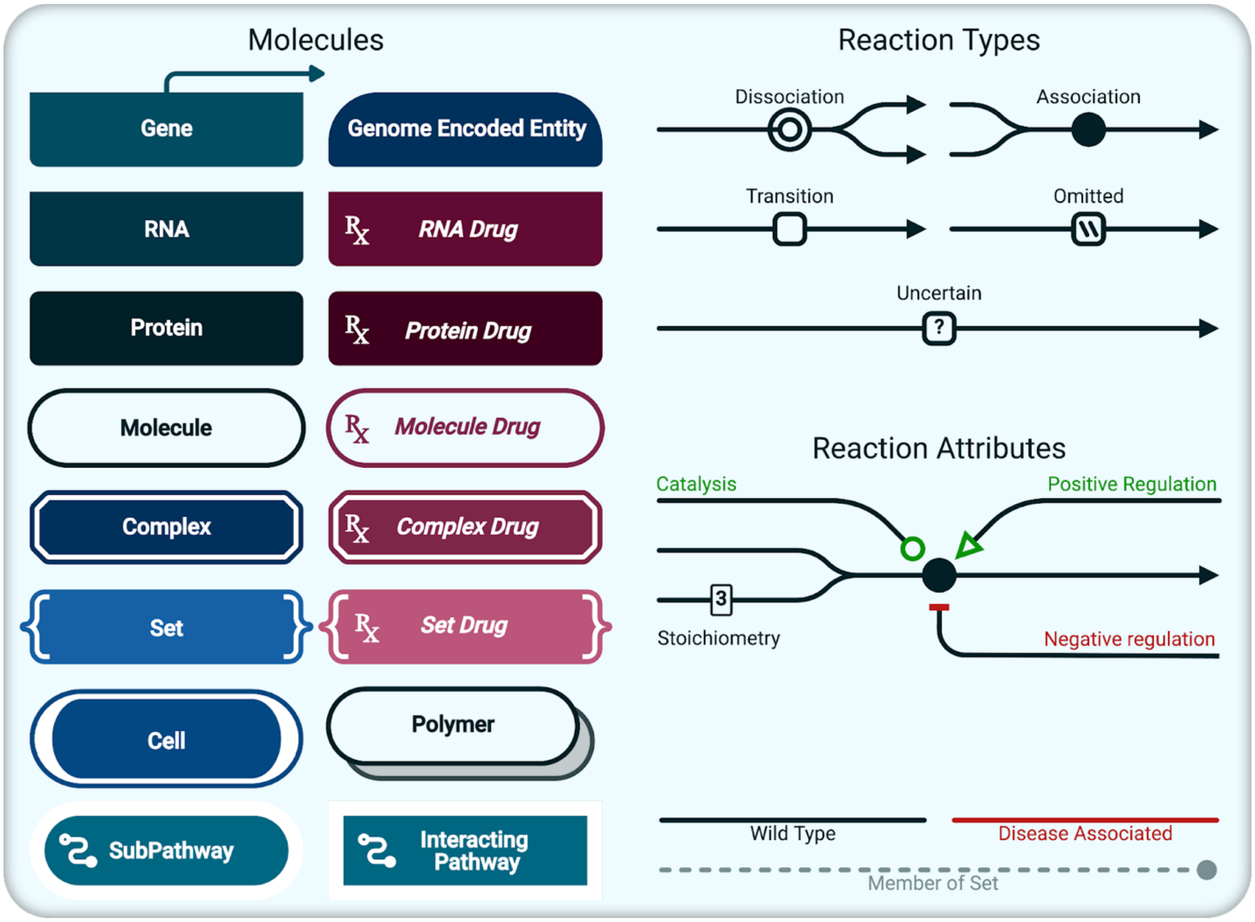

Supplementary Figure 2. **New designs of ELV elements.** Genes, RNAs, and Proteins use the main theme color, with progressively deeper shades along the transcription and translation chain, ensuring that proteins remain visually distinct against the background. Conversely, chemicals are depicted as white capsule shapes outlined in black to maximize visibility. This enhances contrast between these two distinct entity types, which are often displayed together, particularly in metabolic pathways. Entities representing groups of other entities use the secondary theme color and include an inner white rim. Complexes are fully enclosed, as they represent defined groups of molecules in which each member participates. Sets, representing interchangeable reactants, are drawn with a fragmented inner rim forming the symbol of a mathematical set. Cells are also treated as containers for their markers, and therefore use the secondary theme color with an inner white rim. Polymers constitute the only exception: as they consist of a single repeating unit, the graphical representation shows that unit with a diagonal shadow to convey multiplicity. Drugs act as modifiers of entities, denoted by the Rx symbol and recolored according to a dedicated palette. Pathways use the main theme color and include the pathway icon, as they are Reactome-specific.

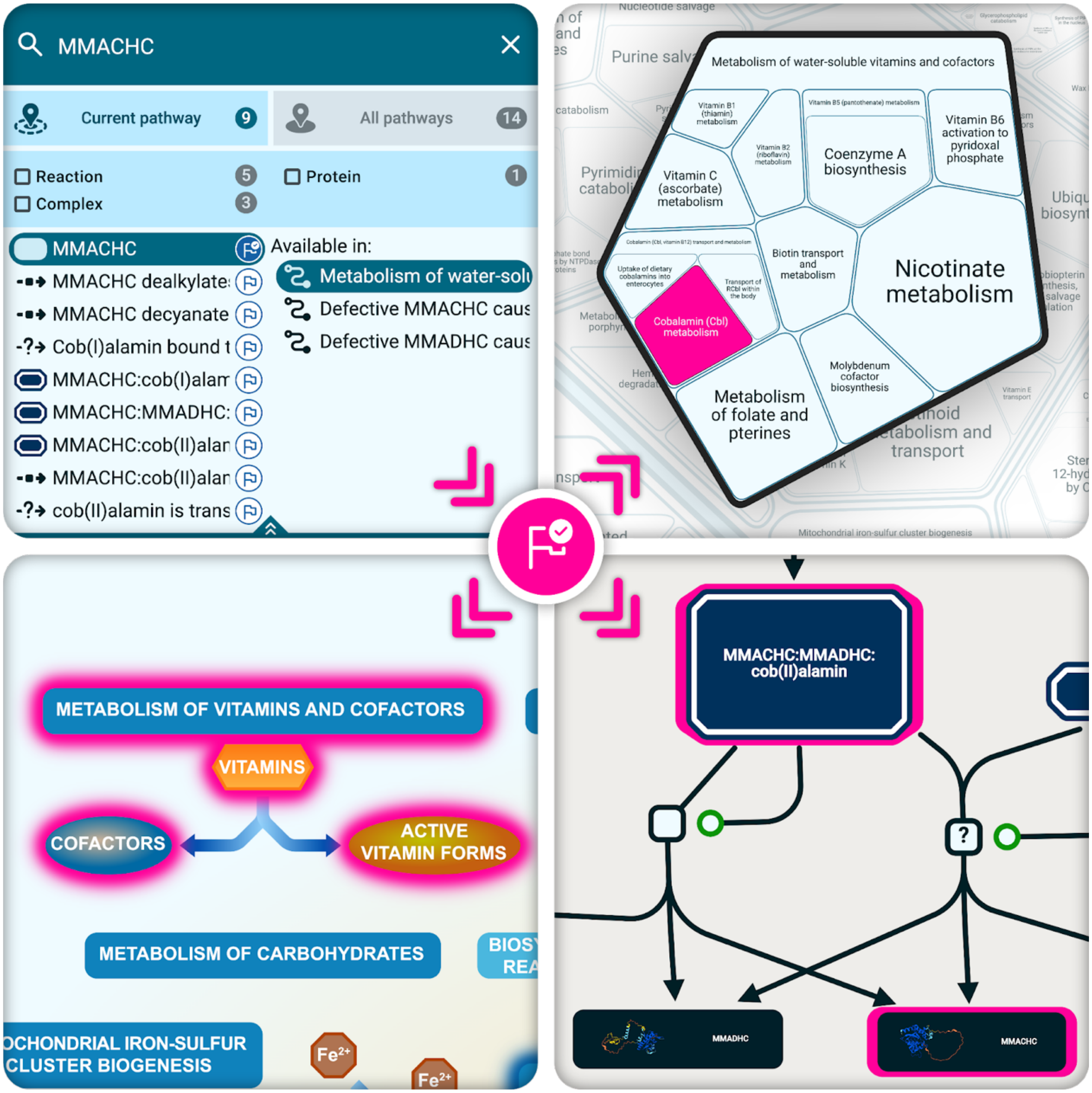

Supplementary Figure 3. Search results and flagging of MMACHC. From left to right and top to bottom: Search results of MMACHC within the "Metabolism of water-soluble vitamins and cofactors" pathway, with MMACHC selected and flagged using the dedicated button; ReacFoam view with MMACHC flagging; Metabolism EHLD view with MMACHC flagging; "Metabolism of water-soluble vitamins and cofactors" ELV with MMACHC flagging

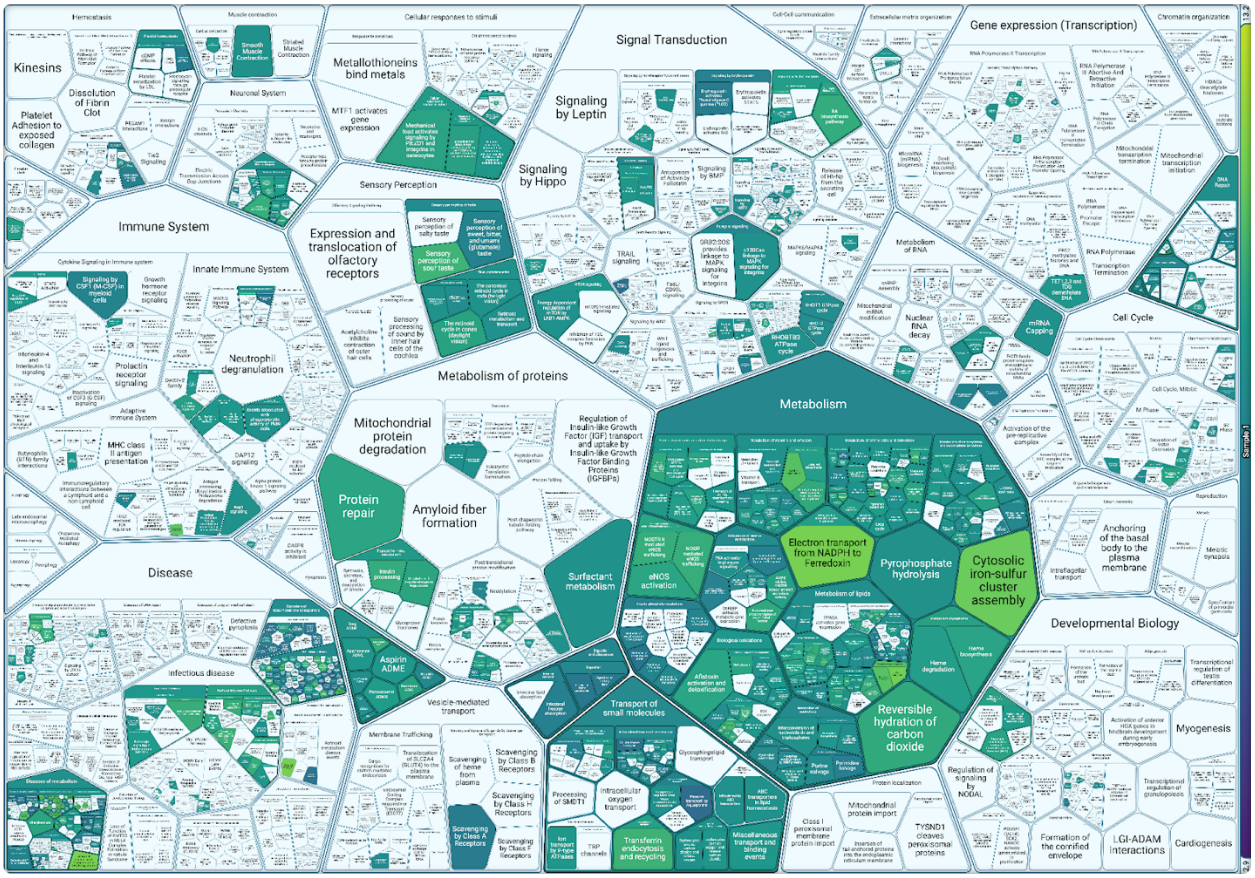

Supplementary Figure 4A **Reacfoam view** **(overview mode).** A qualitative enrichment analysis of the "Metabolomics" example is filtered for FDR < 0.05.

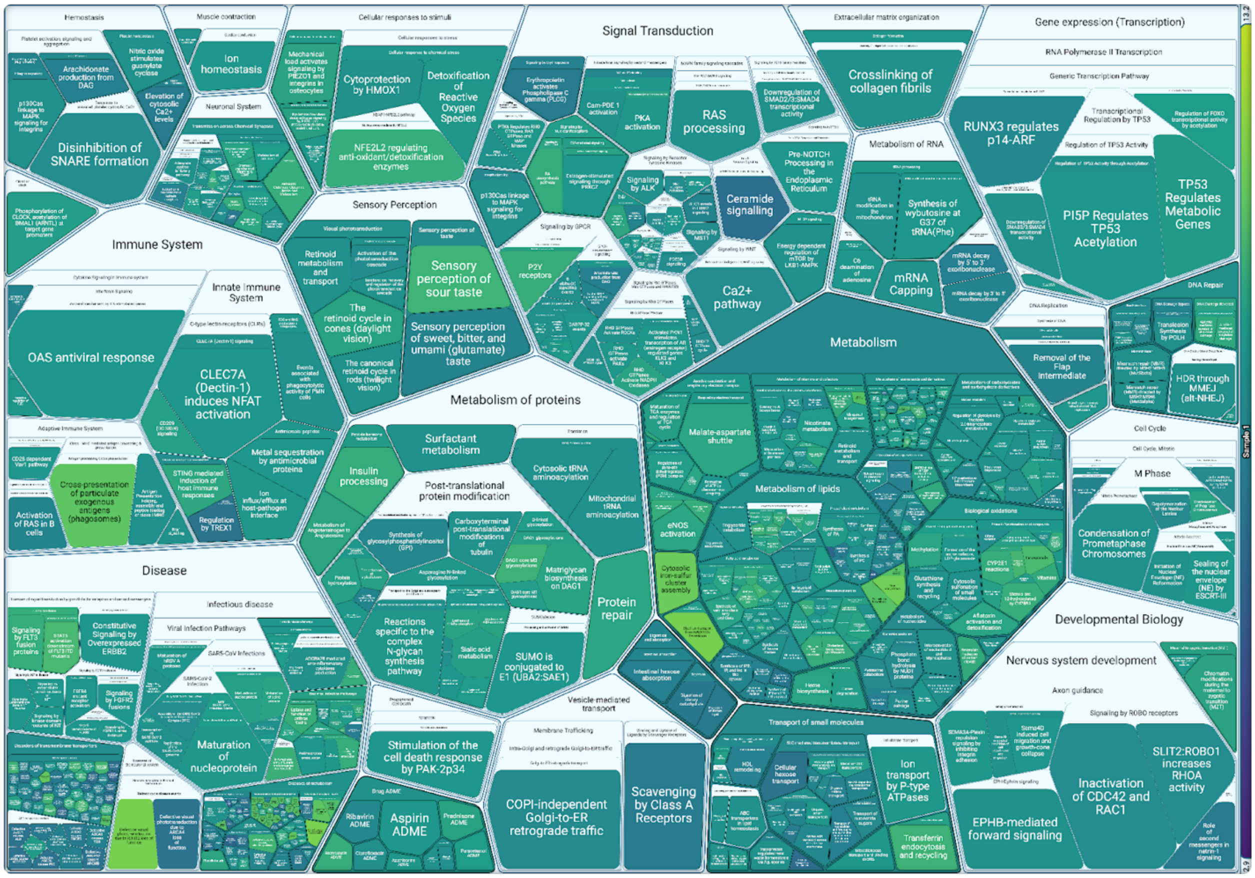

Supplementary Figure 4B **Reacfoam view (focus mode).** A qualitative enrichment analysis of the ["Metabolomics"](Metabolomics) example, filtered for FDR < 0.05.

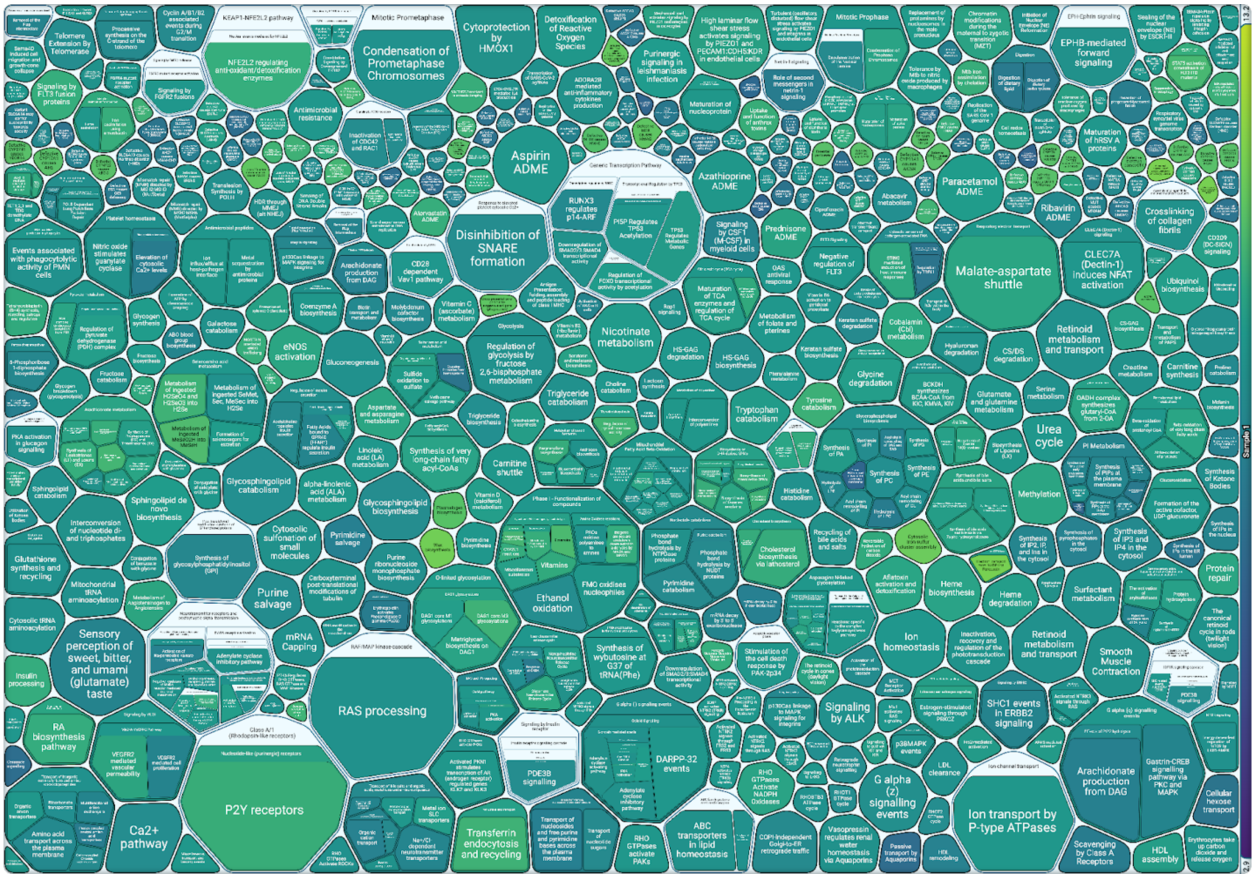

Supplementary Figure 4C **[Reacfoam view](#)** **in focus mode with "Pathway grouping" removed.** A qualitative enrichment analysis of the ["Metabolomics"](#) example, filtered for FDR < 0.05.
